# Section 3. A Discussion of Flexible Dosing and Patient-Centered Therapy: *Highlights of the Asthma Summit 2009: Beyond the Guidelines*

**DOI:** 10.1097/WOX.0b013e3181d27cd8

**Published:** 2010-02-15

**Authors:** G Walter Canonica, Christopher Brightling

**Affiliations:** 1 Allergy and Respiratory Diseases Department, Genoa University, Genova, Italy; 2 Institute for Lung Health, University of Leicester, Leicester, UK

**Keywords:** asthma, inhaled corticosteroid, flexible dosing, combination therapy, long-acting beta-agonist

## Abstract

Despite positive clinical experience and the published clinical benefits of monotherapy with low-or medium-dose inhaled corticosteroids or combination therapy with ICS + long-acting beta-agonist to treat asthma, many patients remain suboptimally controlled. Alternative approaches are needed, and 3 options that have had some success are: 1) using the patient's level of inflammation by established biomarkers to set treatment; 2) self-management incorporating flexible dosing; and 3) using a single inhaler for rescue and maintenance therapy. Which strategy for which patient depends ultimately on the individual patient's disease burden, life-style, comorbidities, preferences, and his or her ability to self-manage the disease, including assessing symptoms and adhering with therapy.

## 

Current recommendations for managing asthma are based on positive outcomes associated with the use of low-dose inhaled corticosteroids (ICS) and higher dose ICS or ICS + long acting beta-agonist (LABA) in combination as recommended at steps 2 and 3, respectively, in both the GINA and EPR-3 guidelines[1,2]. However, many patients remain uncontrolled despite the benefits of treatment demonstrated in clinical trials. Therefore, there is a substantial unmet need. Within this group some patients will have severe asthma, which may be 'difficult-to-treat' or 'refractory' to high doses of ICS or even oral corticosteroids with or without other medications. This group presents a clinical challenge and needs to be managed by an asthma specialist. Control can be regained in some using alternative strategies. Recognizing this, the question is: "What can we do to improve asthma control for these step 2 and step 3 patients."

Alternative approaches are, thus, needed to improve asthma control. Three options that have had some success will be reviewed briefly: 1) treating patients according to their level of inflammation as demonstrated by established biomarkers; 2) flexible dosing for patient self-management; and 3) using a single inhaler.

## Treating patients according to their level of inflammation

**Figure 1:**
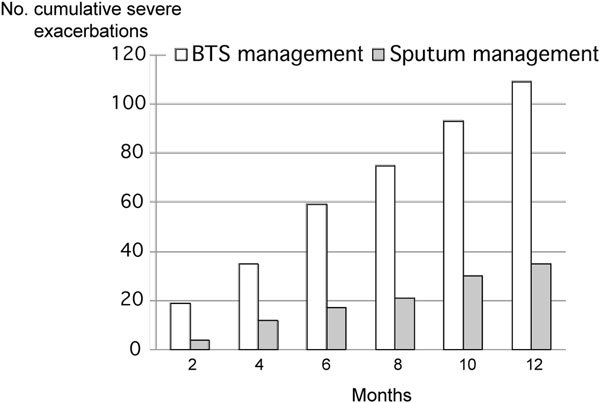
**Cumulative severe exacerbation rate in patients treated according to the British Thoracic Society (BTS) asthma management guidelines (n = 37) or by normalization of induced sputum and reduction in symptoms (n = 37)**. A total of 7 hospital admissions were reported during the 12 month treatment period, 6 in the BTS management group and 1 in the sputum management group (*P *= 0.002) [3].

A controlled study compared patients with asthma who were treated according to standard British Thoracic Society guidelines and patients who were treated based on their level of inflammation as determined by sputum eosinophil counts[3]. Striking differences were reported between the 2 groups, with more patients treated by level of inflammation showing marked reductions in the number of severe exacerbations (Figure 1) and reductions in hospital admissions[3]. This observation has been replicated in 2 further studies and has been confirmed in a meta-anlaysis [3-6].

Sputum-based treatment is now recommended by the British Thoracic Society[7]. However, in these studies the benefit of managing patients based on sputum was limited to those with more severe disease and to those who showed eosinophilia at the time of exacerbation. Patients who have exacerbations related to neutrophilia may respond better to different approaches to therapy, but this requires further study.

## Flexible dosing

Flexible dosing appears to be an effective alternative for asthma management. Intermittent therapy used as needed has been studied in patients with mild asthma. Fixed doses of budenoside given twice-daily as monotherapy or in combination with zafirlukast were superior to intermittent, symptom-guided treatment as measured by prebronchodilator forced expiratory volume in the first second (FEV_1_), exhaled nitric oxide, sputum eosinophilia, asthma control scores, and symptom-free days[8]. However, improvements in quality of life measurements, postbronchodilator FEV_1_, morning PEF, and rates of exacerbations were similar in all treatment groups, suggesting that intermittent therapy could be comparable to fixed-dose ICS[8]. In a study randomizing patients to as-needed albuterol, as-needed beclomethasone dipropionate (BDP), fixed daily BDP/albuterol, or as-needed BDP/albuterol, symptom-driven use of BDP/albuterol was as effective as regular use of BDP, with a lower cumulative dose of ICS over 6 months[9]. A randomized trial of 3400 patients reported that formoterol used as needed provided better protection against severe exacerbations than terbutaline; the addition of budenoside to the as needed formoterol enhanced the reduction in exacerbations [10].

## Using a single inhaler

Use of a single inhaler containing a LABA and ICS combination appears to provide more effective asthma control at lower ICS doses,[11] and for acute symptoms the combination of budenoside/formoterol administered by Turbuhaler has been shown to be well tolerated and as effective a bronchodilator as albuterol administered by pressurized metered-dose inhaler[12]. A meta-analysis of 15 studies representing a total of 15,000 patients found that, with respect to treatment failure defined by hospitalization, use of oral corticosteroids, or withdrawal from a study, patients using the budenoside/formoterol combination in a single inhaler showed better improvement than those using a fixed dose of budenoside or fomoterol[13].

**Figure 2:**
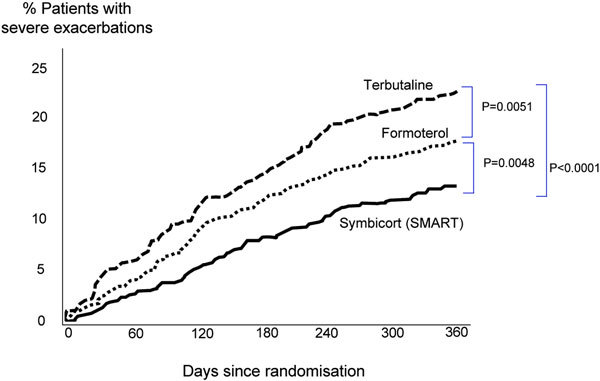
**Time to first severe asthma exacerbation in patients treated with budesonide/formoterol (160/4.5 *μ*g bid, combination) plus as needed therapy with terbutaline (0.4 mg, n = 1138), formoterol (4.5 *μ*g, n = 1137), or the combination (SMART, n = 1107)**. All patients randomized were symptomatic on the maintenance combination therapy during a 2-week run-in. Reprinted from Rabe KF, Atienza T, Magyar P, Larson P, Jorup C, Lalloo UG: Effect of budesonide in combination with formoterol for reliever therapy in asthma exacerbations: a randomized controlled, double-blind study. *Lancet*. 2006;368: 744-753, with permission from Elsevier [10].

Indeed, using budenoside/formoterol (Symbicort) as maintenance and reliever therapy (ie, the "SMART" approach) appears to be particularly effective. In terms of exacerbation burden and time to first severe exacerbation, the SMART approach was favored over the use of budesonide/formoterol plus either terbutaline or formoterol as reliever (Figure 2) and also over fluticasone propionate/salmeterol as maintenance plus terbutaline as reliever[14]. Data from this study also showed that the SMART approach controlled asthma to a similar degree as the combination maintenance plus separate reliever regimens, but at lower overall drug doses. The results were consistent with those from a similar study in which physicians were able to titrate fluticasone propionate/salmeterol to reflect real-life situations [14].

It is not clear why the SMART approach is effective. ICS may modulate airway remodeling as illustrated by decreased smooth muscle, reduced epithelial damage, and inhibition of myofibroblast differentiation after treatment[15,16]. Data from recent studies suggest that adding a LABA enhances these effects[17-21]. Eight of 10 studies in a recent meta-analysis favored ICS/LABA combinations over increased ICS doses as a step-up for patients with mild to moderate asthma,[22] and the synergistic effects of ICS and LABA might also occur in patients with severe asthma [23].

The synergistic effects of ICS and LABA do not distinguish between patients on maintenance therapy and those who also use the combination as a reliever. However, in a survey of 3500 patients, patients using short-acting beta-agonists (SABA) as a reliever adjusted their reliever medications early, when warning signs of an exacerbation occurred, and showed a 4-fold increase in reliever treatment at the time of worst symptoms[24]. Patients using additional ICS doses for acute symptom relief did not show this same magnitude of increase. These data are consistent with those from another study, which suggested that quadrupling the ICS dose, but not doubling it, can shorten the course of an exacerbation [25].

In a study in which the budesonide/formoterol combination was used as maintenance and reliever therapy, superimposed plots of symptoms and peak expiratory flows (PEF) on use of the combination as rescue indicated that, similar to the SABA data described above, patients increased their use of budenoside/formoterol before the onset of their worst symptoms[26]. The data support earlier reports that increasing the combination dosage can prolong time to first severe exacerbation, increase time to medical intervention, and reduce the overall risk of exacerbation[27,28]. Thus, the data suggest that patients using the SMART approach increase their use of medication when they experience warning signs of an impending exacerbation. With the combination therapy the increased dosage of ICS may be sufficient to influence exacerbation frequency. An alternative, though controversial, explanation is that SABA exert negative effects, as suggested by data showing that SABA use reduces asthma control, aggravates airway response to allergen challenge, and increases mast cell mediator release[29,30]. However, a 2007 study found no evidence of such harm associated with SABA [31].

Despite early evidence suggesting a LABA-associated risk for sudden death or exacerbations,[30] a recent study has found no such evidence of when a LABA is used in combination with ICS[32]. These data support the preference for a combination of low-dose ICS and LABA at treatment step 3 in the GINA guidelines [1].

It is clear that for managing the asthma patient, one size does not fit all. Different therapeutic strategies must be weighed against the individual patient's disease burden, life-style, comorbidities, and preferences. This is true regardless of the patient's initial level of severity, and while our focus has been on steps 2 and 3, many of the suggestions are applicable to managing patients at other levels. Guideline recommendations are just that, a guide for therapeutic decision-making. Also important is the patient's phenotype, the ability of the person to judge changes in symptoms, and adherence with treatment.

Most patients seem to prefer flexible dosing approaches. A study of preferences among patients with mild asthma found that the percentage of patients preferring to use beclomethasone and formoterol inhalers on an as needed basis was similar to those preferring once-daily ICS/LABA in a single inhaler; also, more patients preferred either option than the regular use of a leukotriene receptor agonist with albuterol rescue[30]. Data from a small, open-label study across 44 primary care practices also suggested that adherence increases with a single-inhaler approach[33]. Additionally, the costs for single-inhaler therapy are likely to be lower, because patients use less drug overall [34].

**Figure 3:**
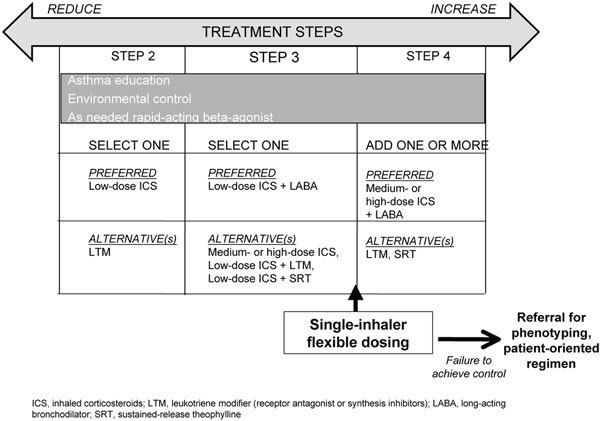
**Proposed modified stepwise approach for managing asthma in patients ≥ 5 years of age with persistent mild or moderate disease, focus on added step between 3 and 4 (adapted from GINA)**.

At present there is no simple means to phenotype patients with asthma. Biomarkers are not yet sophisticated enough and economic justification is not yet clear enough to apply this approach to all patients with asthma. For now, it may simply be preferable to prescribe a single-inhaler, flexible dosing approach as a step-up between steps 3 and 4 (Figure 3). Patients who do not achieve asthma control on this regimen could then be referred to difficult-asthma services and care, with more detailed phenotypic analysis. In this scenario, careful phenotyping would be restricted to patients with more severe disease and greater disease burden, where the expense could be better justified. This would be a truly patient-centered therapeutic approach.

## Discussion

**Dr. Oppenheimer: **My question is, "How can we really do this in our practices?" In the US, if we give our patients permission to do this and something goes wrong, the Food and Drug Administration (FDA) will be on the side of the plaintiff.

**Dr. Bukstein: **I agree it's a problem, but I think with good communication and proper documentation, it can be overcome. The key is communication with patients. I tell my patients that this is not FDA-approved, but it is a form of therapy that I think might be effective for them. Then I give them a choice of therapy, and I document the conversation, that I explained the risks and benefits to the patient and that they made their decision based on their individual needs. There are many things that may not be approved but are still the best practice for that individual patient.

**Dr. Brightling: **I agree. What's absolutely key with a combination inhaler is that you can overcome the concern about using LABA in the absence of ICS.

**Dr. Kaliner: **Dr. Oppenheimer, the question is a good one and one we face every day in practice. As the asthma experts in our communities, where is the fear of trying something new? The fear is lawsuits or reputation. We set the standard of care for treating asthma. The guidelines provide general rules, but they are only guidelines; you have to add your personal experience. I think most patients are really thrilled when we try new things. It's our job to lead the pack and not to wait for someone 10 years from now to finally recognize that this works.

**Dr. Luskin: **Dr. Canonica, how comfortable should we be with formoterol and its safety, particularly when we're offering our patients the option to use it as rescue therapy?

**Dr. Canonica: **First, let me say that in southern Europe we don't have so many problems with the LABAs. It is mainly a US issue.

**Dr. Luskin: **We heard that formoterol is an "improved LABA" and that the presence of the ICS protects against any potential adverse effects because of the LABA. Does the data support that?

**Dr. Bousquet: **The published data suggested that ICS may protect against the adverse events observed when LABA were used alone. However, I don't think this has been proven by subsequent studies, despite the comments of the December 2008 panel at the FDA. For me, there are no real side effects of LABA, and this debate is dangerous because if we stop LABA, we'll have more deaths from asthma.

**Dr. Bukstein: **We need to get patients to understand that the burden of asthma therapy really is not very high. The predominance of data did not show that LABA are harmful. If patients are overly concerned about side effects, they are going to make the wrong decision and won't take the medication.

**Dr. Luskin: **So, for the formoterol-ICS combination products, should we be comfortable with regular use plus as-needed use; 1, 2, or maybe 3 doses more during a day?

**Dr. Bousquet: **First, remember that the problem initially was the dose of ICS used and not the dose of LABA. Second, studies have shown that formoterol is safe at doses up to 54 *μ*g. There are early adverse effects (eg, tremor and tachycardia) that are probably not so important; there are also long-term effects, including serious exacerbations, even deaths. However, these have occurred mostly in patients using LABA as monotherapy, not with combination therapy.

Finally, I think that we have to be very careful not to say to patients, "This is dangerous," because in reality deaths due to asthma have decreased considerably, particularly with use of combination therapy.

**Dr. Brightling: **We also need to think about when to move on to the other paradigm for the individual patient. For me, that is when the patient is not getting good control with the flexible dosing approach. At that point you need more intensive investigation and, maybe, more continuous treatment under the management of an asthma specialist.

**Dr. Canonica: **In Italy, the initial proposal was to use the budesonide/formoterol combination bid plus prn up to 2 additional doses if needed. The concept then was to reduce dosing, but because of the costs of the treatment and not because of side effects. Sometimes we have to look at the reality from different perspectives.

**Dr. Spector: **I'd like to ask about another type of flexible dosing that wasn't brought up; namely, using high doses of ICS to treat exacerbations. At least from pediatric studies (eg, Rodrigo et al) it seems that that's another alternative for getting over an exacerbation successfully without using a LABA.

**Dr. Bukstein: **But, the ICS doses suggested are high enough to get systemic absorption, so why not just use oral corticosteroids?

**Dr. Spector: **Well, it's got the advantage that Dr. Brightling was talking about, the patient is using the same inhaler; and, presumably, only for a short period of time, to get them over an episode. I'm bringing it up because I don't think it's been studied very well, and maybe it should be a study for the future.

**Dr. Luskin: **Does the panel agree that the mechanism of success of SMART, assuming that SMART actually works, is timing? That's the theory, that we're really introducing doses of ICS earlier than we might otherwise; and doubling the ICS dose doesn't seem to work. So, is this all timing or are there other nongenomic effects?

**Dr. Bukstein: **Maybe it's better adherence with once-a-day dosing and carrying only one inhaler.

**Dr. Bousquet: **Most patients do what they want. We should remember that. So with a single inhaler, the approach becomes very simple. They use what they need. The only warning that I give my patients is to say that if they need to use their inhaler 4 times a day or more for longer than 3 days, they need to contact me. This is a safety issue.

**Dr. Calhoun: **There are 2 LABA regulatory issues here. One is using LABA in asthma, as in the SMART study; the other is the use of the LABA, formoterol, in a maintenance and rescue kind of SMART study. As Dr. Bousquet said, the evidence that LABAs are harmful in asthma is trivial. That question was asked and answered definitively at the December 2008 FDA meeting, and unless new data arise regarding safety, I don't think that question will come up again. The issue about maintenance and relief might be different based on the framework in which the FDA operates. Their mandate is to establish that drugs on the US market are safe and effective. For a combination product, the individual components must be shown to contribute to the overall efficacy. This is not a problem for maintenance; but it is more difficult to show improvement on the reliever side of the equation. The reliever effect of formoterol is pretty clear. The benefit of adding the ICS, however, is not so clear. Synergy has not been shown clinically in terms of quick relief. Thus, you must convince the agency that using the combination cuts down on the need for more reliever.

However, the rigid framework of the FDA does not necessarily need to constrain physicians who are operating at the cutting edge. not doing things that are out of the mainstream. We're not putting patients' safety at risk, so as long as the patient is aware that this is technically off-label. I think Dr. Kaliner's approach is correct. We ought to be pushing the envelope just a little bit, as long as it leads to better patient care.

**Dr. Oppenheimer: **It needs to be put in the framework of higher than recommended doses in the US.

**Dr. Calhoun: **Yes, we need to be careful how we use the rescue piece because formoterol is clearly where the toxicity is. So, we need to have an upper limit of the number of puffs and some discussion with the patient that is documented.

**Dr. Luskin: **Dr. Bousquet uses more than 4 times a day for 3 days, for a phone call. How does the panel feel about that?

**Dr. Bukstein: **I use a similar approach: more than 4 times a day for 2 consecutive days requires a phone call to me. Interestingly, we almost never get phone calls from patients doing that; they tend to control their disease fairly well.

**Dr. Brightling: **We also have a similar approach and do not get many phone calls from patients using single inhaler therapy.

**Dr. Canonica**: I agree, we have the very same experience.

**Dr. Kaliner: **This is a fine set of suggestions, but I would add peak flow measurements. We tell our patients to contact the office at a certain point of peak flow drop (eg, 40%). That's our other way of determining how severe their problem is.

**Dr. Hargreave: **I have 2 differences of opinion. First, I don't think that we have the evidence that doubling the dose of ICS at the start of an exacerbation does not work. I have a different interpretation of that study. The subjects, who had been followed over a long period of time, were on ICS and had an exacerbation that lasted for about 2 weeks. The placebo and active groups were similar, which says to me that this was a viral noneosinophilic exacerbation, which would not be expected to respond to steroid. I don't think we can do these types of studies unless we also do inflammatory measurements to see if the exacerbation is steroid responsive.

**Dr. Luskin: **This is important. We know it's proven that ICS are the best drugs of first choice early on; but we also know that one size doesn't fit all. Going back to the idea of SMART therapy and looking at the data, there clearly is a benefit to maintenance and reliever therapy with one combination inhaler. But, who are the responders? Who are the candidates for this type of approach?

**Dr. Bukstein: **I usually present SMART therapy as an option to someone who I think can comply with it, a patient who can judge their own symptoms, peak flows and know when they are having increasing problems. In other words, it is the patient who has good insight into his or her disease who is appropriate for this approach. A few PCPs that do this have told me that this approach works so well that if a patient is failing, that's a signal for referral. It indicates more complex problems.

**Dr. Luskin: **So, we need to add that if this approach doesn't work, there should be a specialist referral.

**Dr. Bousquet: **I agree.

**Dr. Brightling: **That underscores exactly my presentation. Ideally, it would be great to try to understand the triggers before defining treatment; but at this point in time we don't have simple ways to do that. It is simpler to give treatment and then, if the patient fails, go back and try and understand the disease, the triggers, and the phenotypes.

**Dr. Luskin: **Is there a clinical phenotype that helps you determine whether or not this patient is an appropriate candidate?

**Dr. Brightling: **The presence of eosinophilia is a good predictor of response to ICS. Where we struggle are the patients who don't have eosinophilic inflammation because we don't have good therapies for them. But, if you're going to give combination therapy up front anyway, then you could argue against measuring the inflammation in the beginning. You just treat the patient.

**Dr. Kaliner: **So, it depends on what kind of patients you actually see. The level 2 and 3 patients are easy to treat, and that would be the place where this approach fits without any problems. But I usually see patients who are level 4 and higher. These patients first have to be stabilized at a much greater degree of control.

**Dr. Bukstein: **Another problem with using the formoterol/ICS inhaler as a reliever is that it is new and different approach. So, the patient who maybe goes to the pharmacist or to another physician or to the emergency room is told, "You shouldn't do that. Here's the inhaler that you should use to move up and down." And patients usually follow their most recent instructions.

**Dr. Luskin: **Also, if a patient is on bid combination therapy (which is a month's worth) but uses it extra for rescue, is there problems getting a refill?

**Dr. Bukstein: **Huge problems; you almost have to supplement them with samples.

**Dr. Hargreave: **For the majority of doctors who have a short time to see a patient, I have no issue with the SMART approach. One inhaler is fine. But one of the problems I have with guidelines is that specialists, who have more time and have the ability to phenotype patients and to understand what's going on, yet don't do these things because they follow the guidelines. That's wrong. For example, a lot of people that I see just need a certain dose of ICS. Once symptoms are controlled, they don't need a short-acting bronchodilator, so, why should they be given a long-acting bronchodilator? And this isn't a small number of patients. I would caution against everybody using the SMART approach. I think as specialists we need to be a bit more aware of the other approaches.

**Dr. Bousquet: **That would be nice if patients were compliant. Many of the serious asthmatics I see are severe because they don't take their drugs.

**Dr. Bukstein: **I agree with Dr. Bousquet. The optimal approach would definitely be to control somebody on a small daily dose of ICS, but that doesn't occur as often as we'd like.

**Dr. Kaliner: **We're really talking about a new concept in asthma treatment. We were taught that using ICS regularly would prevent remodeling and exacerbations, and we advocated that approach despite the fact that our patients didn't use their medications regularly. The studies about prn ICS and the SMART approach are opening up new opportunities for managing our patients. Dr. Hargreave is saying that level 2 patients don't generally need a combination product and shouldn't be using one. They should use the lowest level of ICS and then increase the dose as needed for exacerbations. However, let's be clear, with the guidelines and the data about new products and approaches such as flexible dosing, we are evolving new paradigms. This discussion is truly important, and that is a key purpose for this meeting.

## Note

G. Walter Canonica, MD, received grants/research support from ALK, Abellò, Stallergenes, Lofarma, AstraZeneca, Schering Plough, Chiesi, Merck, Sharp & Dohme; he is a consultant for ALK Abellò, Stallergenes, Lofarma, AstraZeneca, Schering Plough, Chiesi, Merck, Sharp & Dohme; on the speakers' bureau for ALK, Abellò, Stallergenes, Lofarma, AstraZeneca, Schering Plough, Chiesi, Merck, Sharp & Dohme. Christopher Brightling, MD, received grants/research support from AstraZeneca, MedImmune, GlaxoSmithKline (GSK), and is a Wellcome Senior Clinical Fellowship.
